# Exogenous Expressions of FTO Wild-Type and R316Q Mutant Proteins Caused an Increase in HNRPK Levels in 3T3-L1 Cells as Demonstrated by DIGE Analysis

**DOI:** 10.1155/2017/8216180

**Published:** 2017-05-07

**Authors:** Nil Guzel, Murat Kasap, Aylin Kanli, Gurler Akpinar, M. Dogan Gulkac, Kubra Karaosmanoglu

**Affiliations:** ^1^Department of Medical Biology, Kocaeli University Medical School, Umuttepe, 41380 Kocaeli, Turkey; ^2^Department of Biomedical Engineering, Technology Faculty, Kocaeli University, Umuttepe, 41380 Kocaeli, Turkey

## Abstract

Fat mass and obesity-associated protein is an enzyme that oxidatively demethylates DNA. Although there are numerous studies regarding the catalytic function of FTO, the overall existence or absence of FTO on cellular proteome has not been investigated. This study investigated the changes in the soluble proteome of 3T3-L1 cells upon expression of the WT and the mutant (R316Q) FTO proteins. Protein extracts prepared from 3T3-L1 cells expressing either the WT or the mutant FTO proteins were used in DIGE experiments. Analysis of the data revealed the number of spots matched to every member and there were 350 ± 20 spots with 30.5% overall mean coefficient of variation. Eleven regulated protein spots were excised from the gels and identified by MALDI-TOF/TOF. One of the identified proteins was heterogeneous nuclear ribonucleoprotein K, which displayed more than 2.6- and 3.7-fold increases in its abundance in the WT and the mutant FTO expressing cells, respectively. Western blot analysis validated these observations. This is the first study revealing the presence of a parallel increase in expressions of FTO and HNRNPK proteins. This increase may codictate the metabolic changes occurring in the cell and may attribute a significance to HNRNPK in FTO-associated transformations.

## 1. Introduction

Fat mass and obesity-associated protein (FTO) is an enzyme known to catalyze Fe (II)- 2-oxoglutarate-dependent demethylation in both ssDNA and RNA [1–3]. Since the discovery of the FTO gene in a genome wide association study (GWAS) in 2007, numerous efforts have been placed on understanding the physiological function of the FTO. FTO is able to catalyze 3-methyl thymine to thymine and 3-methyl uracil to uracil in ssDNA and RNA, respectively [3]. This reversible activity is interpreted as part of a nucleic acid repair mechanism [4]. However, in a recent study, N^6^-methyladenosine is shown to be a better suited substrate for the FTO indicating that FTO may possess a physiological function different than the predicted one [2]. FTO can oxidatively demethylate the methyl group and change it to short lived mRNAs including N^6^-hydroxymethyladenosine and N^6^-formylmethyladenosine in the nucleus [5].

The clinical significance of FTO was recognized in earlier studies when researches deleted a FTO containing region from the mouse genome and studied its phenotypic effects. What they observed was that there were a partial syndactyly, thymic hyperplasia, growth retardation, and serious malformations in craniofacial structure. Even death was an outcome during the early embryonic stage [6, 7]. Later detailed studies established a direct link between FTO and obesity, which ends up producing a devastating disease, diabetes [8, 9].

Several of the mutations occurring on the FTO gene and their effects on the FTO protein were characterized in detail [10, 11]. The mutations generally affect the activity of the enzyme. For example, the R316Q mutation causes complete loss of the enzyme activity [1, 12]. The phenotypic effects of R316Q mutation were studied using fibroblast cells isolated from a patient with an autosomal-recessive lethal syndrome [12]. In comparison to the control cultures, the R316Q mutant FTO expressing cells displayed altered cell morphology, enlarged cell size, decreased cell viability, and increased senescence [12]. However, the molecular details occurring in fibroblast cells carrying the mutant FTO protein are not known in detail. In this study, we sought to search for the changes occurring at the molecular level using a proteomics approach in 3T3-L1 cells expressing the mutant FTO protein. 3T3-L1 cells are adipose tissue-derived cells, displaying fibroblast-like morphology. As a control, we used the WT FTO-expressing 3T3-L1 cells and performed a detailed comparative study. Our hypothesis was that exogenously expressed mutant FTO protein might cause changes at the proteome level in 3T3-L1 cells that would shed some light onto the observed phenotype. Recent studies suggested that m6A is a key molecule and its modification by FTO creates a code for the regulation of gene expression and in turn protein translation [2, 13].

The proteome of 3T3-L1 cells did not display pronounced changes following either the WT or the mutant FTO expressions. We detected minor changes in abundance of 11 proteins. However, after implementation of strict twofold regulation criteria, there were two proteins left that were significantly regulated. Those were heterogeneous nuclear ribonucleoprotein K (HNRNPK) and Vimentin. HNRNPK is a multifunctional mRNA-binding protein and Vimentin is one of the class-III intermediate filaments found in various cell types. Vimentin, which is listed as one of the most frequently identified proteins in proteomics studies, was disregarded in our further analysis. More of the focus was placed on HNRNPK to elucidate its association with FTO.

## 2. Materials and Methods

### 2.1. Creation of Plasmid Constructs

The full-length cDNA of the human FTO gene was cloned into pcDNA4/TO (Life Tech, USA) by reverse transcription. The pcDNA4/TO clone for the mutant FTO (R316Q) was created in our laboratory by Site-Directed Mutagenesis using a commercial kit (Invitrogen, USA). The constructs were sequenced and in-frame FTO sequences were verified (Eurofins, DE).

### 2.2. Expression of the WT and the Mutant FTO Proteins

3T3-L1 cells were grown in DMEM supplemented with 10% (Vol/Vol) fetal bovine serum, 100 U/ml penicillin-streptomycin, and 2 mM L-glutamine at 37°C in a humidified 5% CO_2_ atmosphere. Cells were grown in Petri dishes to 60% confluence. To express FTO in 3T3-L1 cells, pcDNA4/TO harboring FTO gene sequences was transfected using Lipofectamine 3000 (LifeTech, USA). Empty pcDNA4/TO transfected cells were used as a negative control.

### 2.3. Fluorescence Microscopy

3T3-L1-WT and mutant FTO expressing cells were cultured under standard tissue culture conditions. Culture plates contained glass cover slips that allowed fluorescence imaging. After 48 hrs of FTO expression, cells were fixed with formaldehyde and stained for FTO with an anti-FTO antibody (Clone C-3 (sc-271713), Santa Cruz, USA). The nuclei of the cells were stained with DAPI (Lifetech. USA). Coverslips were mounted in Mowiol before the analysis [14]. Imaging was performed with an inverted microscope (Olympus CKX41) using appropriate filter sets.

### 2.4. Western Blot Analysis

Cell-free extracts were prepared as described by Akpinar et al. (2014) [15]. Western blot analysis was performed using anti-FTO (Clone C-3 (sc-271713), Santa Cruz, USA), anti-HNRNP K (D-6 (sc-28380), Santa Cruz, USA), and anti-beta actin (Santa Cruz, USA; ACTBD, sc-81178) antibodies as described by Kasap et al. (2015) [16]. Untransfected 3T3-L1 cells were used to determine the level of endogenous FTO expression.

For the purpose of band analysis, ImageJ, a freely available software was used as described by Kasap et al. [17]. The software is available at the National Institutes of Health and can be obtained from http://rsb.info.nih.gov/ij/download.html. ImageJ measures the integrated density of each band by outlining it and using the Analyze/Measure command. The values obtained were plotted with Microsoft Excel software.

### 2.5. Minimal Protein Labeling for DIGE

Cells from three biologic replicates were used for protein extraction. The extracts were prepared in minimal DIGE lysis buffer (7 M Urea, 2 M Thiourea, 30 mM Tris, and 5 mM Mg(CH_3_COO)_2_, pH9) and equal amounts of proteins were pooled to create three different pools which were labeled with Cy2, Cy3, or Cy5 according to the instructions provided by the supplier (Life Tech, USA). The whole labeling experiments were carried out in the dark.

In short, 50 *µ*g of protein sample was used in each labeling experiment. After adjusting the pH of the extracts to 8.5, CyDye DIGE minimal dyes were added directly to the samples and incubated at 4°C for 30 min. The reactions were stopped by adding 10 mM lysine. Labeled samples were then pooled and used in 2D experiments.

### 2.6. 2D Gel Electrophoresis Experiments

For DIGE experiments, three sets of gels were produced. The labeled protein samples were loaded onto immobilized 11 cm, pH gradient strips (IPG) (pH 3–10) by passive rehydration. Separation based on isoelectric points was performed by a Protean Isoelectric Focusing Cell (Bio-Rad, USA). The following conditions were used for IEF: twenty min at 250 V with a rapid ramp followed by 2 hrs at 4000 V with a slow ramp and 2.5 hrs for 4000 V with a rapid ramp until a total of 32000 V/h was reached at 20°C. After isoelectric focusing, the strips were washed with buffer I (6 M Urea, 375 mM Tris-HCl pH 8.8, 2% SDS, 20% glycerol, 2% (w/v) DTT) for 30 min and then with buffer II (6 M Urea, 375 mM Tris-HCl pH 8.8, 2% SDS, 20% glycerol, 2.5% iodoacetamide (w/v)) for 30 min at room temperature in the dark and subjected to SDS-PAGE using TGX precast gels in a Dodeca gel running system (Bio-Rad, USA). In parallel to the DIGE experiment, a preparative 2D gel experiment was run to cut protein spots for identification. A total of 240 *µ*g of protein (80 *µ*g from each sample) was loaded for each separation.

### 2.7. Image Analysis

DIGE images were captured using appropriate filter sets with VersaDoc4000 MP (Bio-Rad, USA). PDQuest Advance 2D-analysis software (BioRad, USA) was used for comparative analysis of protein spots. Quantity of each spot was normalized by linear regression model. The statistical significance of image analysis was determined by Student's* t*-test (statistical level of *p* < 0.05 is significant). Gel spots significantly differed in expression (more than 2-fold) and were selected and excised from gels using ExQuest Spot cutter (Bio-Rad, USA) for protein identification. A manual editing tool was used to inspect the determined protein spots detected by the software. The 3D view of the selected spots for each group was created to perform visual comparison using PDQuest Advance 2D-analysis software. The software is run with default settings.

### 2.8. Protein Identification

Protein identification experiments were performed at Kocaeli University DEKART proteomics laboratory (http://kabiproteomics.kocaeli.edu.tr/) using ABSCIEX MALDI-TOF/TOF 5800 system. In-gel tryptic digestions of the proteins were performed using an In-gel Digestion Kit following the recommended protocol by the manufacturer (Pierce, USA). Before deposition onto a MALDI plate, all samples were desalted and concentrated with a 10 *μ*L ZipTipC18 following the recommended protocol (Millipore, USA). Peptides were eluted in a volume of 1 *μ*L using a concentrated solution of *α*-cyano-4-hydroxycinnamic acid (*α*-CHCA) in 50% acetonitrile and 0.1% trifluoroacetic acid in water and spotted onto the MALDI target plate. The TOF spectra were recorded in the positive ion reflector mode with a mass range from 400 to 2000 Da. Each spectrum was the cumulative average of 200 laser shots. The spectra were calibrated with the trypsin autodigestion ion peaks *m*/*z* (842.510 and 2211.1046) as internal standards. Ten of the strongest peaks of the TOF spectra per sample were chosen for MS/MS analysis. All of the Peptide Mass Fingerprints (PMFs) were searched in the MASCOT version 2.5 (Matrix Science) using a streamline software, ProteinPilot (ABSCIEX, USA), with the following criteria: Swissprot; species restriction to* M. musculus *and* R. norvegicus*; enzyme of trypsin; at least five independent peptides matched; at most one missed cleavage site; MS tolerance set to ±50 ppm and MS/MS tolerance set to ±0.4 Da; fixed modification being International carbamidomethyl (Cys) and variable modification being oxidation (Met); peptide charge of 1+; and being monoisotopic. Only significant hits, as defined by the MASCOT probability analysis (*p* < 0.05), were accepted. Protein score is −10*∗*log⁡(*p*) where *p* is the probability that the observed match is a random event. Protein score, which has a value of *p* < 0.05, is considered significant hit. Protein scores are derived from ion scores as a nonprobabilistic basis for ranking protein hits.

### 2.9. STRING Analysis

The STRING database (http://string-db.org) aims to provide a critical assessment and integration of protein-protein interactions, including direct (physical) as well as indirect (functional) associations. STRING analysis was performed at http://string-db.org.

## 3. Results

### 3.1. Endogenous FTO Expression in 3T3-L1 Cells

**Figure 1:**
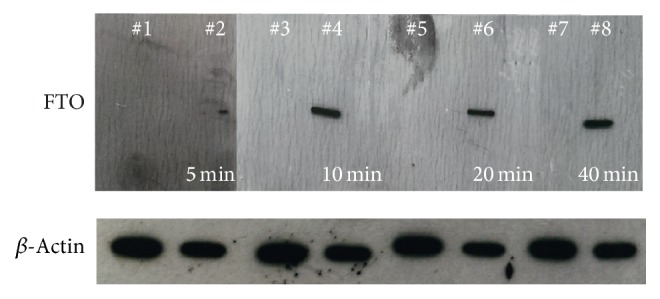
Western blot analysis of endogenously expressed FTO protein in 3T3-L1 (Lanes #1, #3, #5, and #7) and the WT FTO transfected SH-SY5Y control cells (Lanes #2, #4, #6, and #8). For detection of FTO and actin bands, mouse monoclonal anti-FTO and anti-actin antibodies were used, respectively. The FTO band was only observed in the WT FTO transfected SH-SY5Y cells. The lack of FTO band in 3T3-L1 cells implies the absence or low level of FTO expression.

Before the expression of exogenous FTO proteins in 3T3-L1 cells, we measured the expression of endogenous FTO using western blot analysis. A western blot was created using consecutive protein loads of 5 *µ*g/lane from a cell-free extract of 3T3-L1 cells. The blot was cut into four pieces and each piece was probed with an anti-FTO antibody. To visualize endogenous FTO expression, X-ray films were exposed to the blots and different exposure times ranging from 5 min to 40 min were used. There was no distinctive endogenous FTO band appearing on the blots indicating that 3T3-L1 cells did not express FTO protein (Figure 1). The control band appeared on the blots came from SH-SY5Y cells expressing FTO. However, if more than 15 *µ*g total protein was loaded to the gels, a very faint FTO band can be visualized within 20 min of exposure indicating that a low level of FTO expression was present in 3T3-L1 cells.

### 3.2. Exogenous FTO Expression in 3T3-L1 Cells

**Figure 2:**
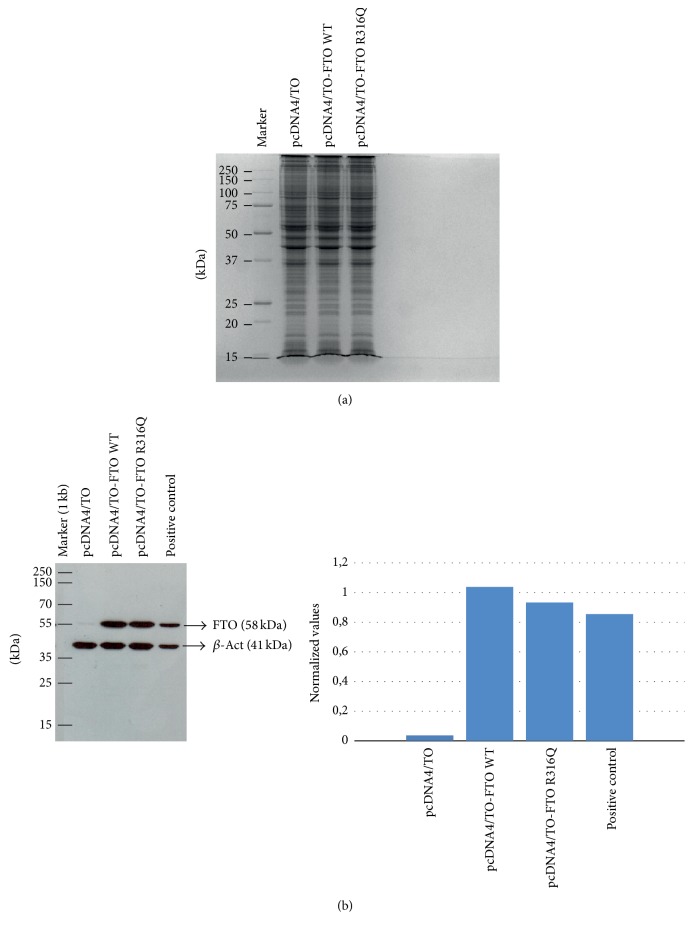
SDS-PAGE analysis of the cell-free extracts prepared from the pcDNA4/TO, pcDNA4/TO-WT FTO and pcDNA4/TO-R316Q FTO transfected cells to demonstrate the moderate expression level of the FTO (a). Western blot analysis of the WT FTO and the mutant (R316Q) expressions in 3T3-L1 cells. The positive control was from the WT FTO transfected SH-SY5Y cell-free extract. The bar graph was created using the intensity values of the bands obtained from Image J.

3T3-L1 cells were transfected with pcDNA4/TO itself or pcDNA4/TO harboring the WT and the mutant FTO genes. After 48 hrs of expression, FTO levels were monitored using SDS-PAGE (Figure 2(a)). There was no overexpressed band indicating that FTO proteins were expressed at a moderate level which would not cause toxic effects on physiological events due to overexpression. To demonstrate FTO expressions, western blot analysis was also performed with the same cell-free extracts used in SDS-PAGE analysis. There was an increase in FTO levels, indicating the success of the transfection (Figure 2(b)).

### 3.3. Fluorescence Imaging of FTO

**Figure 3:**
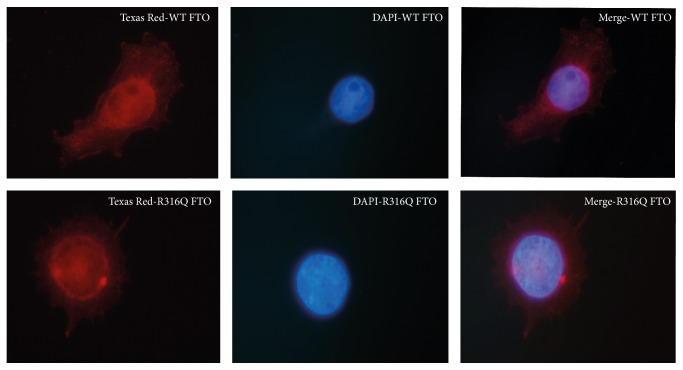
Immunofluorescence analysis of the WT and the mutant FTO-expressing 3T3-L1 cells. The images clearly demonstrated nuclear localizations of the FTO proteins.

To observe the localization of both the WT and the mutant FTO in 3T3-L1 cells immunofluorescence staining was performed. Superposed images were generated using ImageJ software. A similar nuclear localization was observed for the WT FTO and the R316Q mutant proteins in the cells (Figure 3).

### 3.4. 2D-DIGE Analysis of the WT and the Mutant FTO Expressing 3T3-L1 Cells

**Figure 4:**
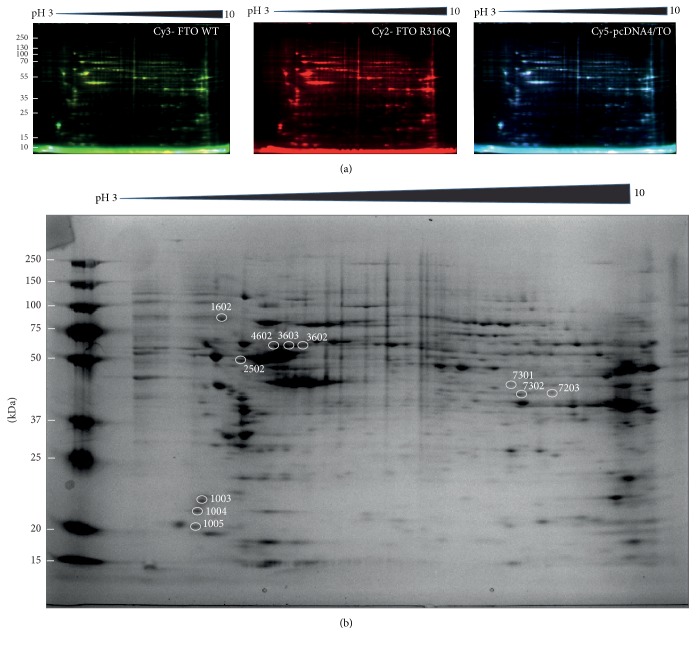
Representative 2D images of the DIGE gels (a). The green color represents Cy3 labeled protein extracts prepared from the WT FTO expressing cells. The red color represents Cy2 labeled protein extracts prepared from the mutant (R316Q) FTO expressing cells. The blue color represents Cy5 labeled protein extracts prepared from the pcDNA4/TO expressing cells. The Image for the 2D preparative gel (b). The spots labeled with SSP numbers were excised and identified.

**Figure 5:**
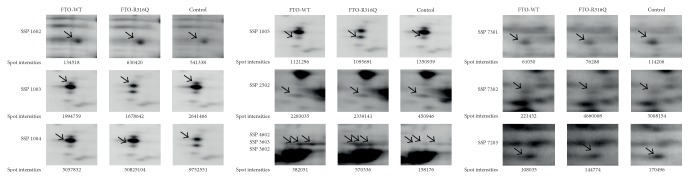
Close-up images of the regulated protein spots and their corresponding spot intensities. The arrows point to the exact position of the spots. Relative quantification of the spots was performed using PD Quest Advanced software (BioRad, USA).

**Figure 6:**
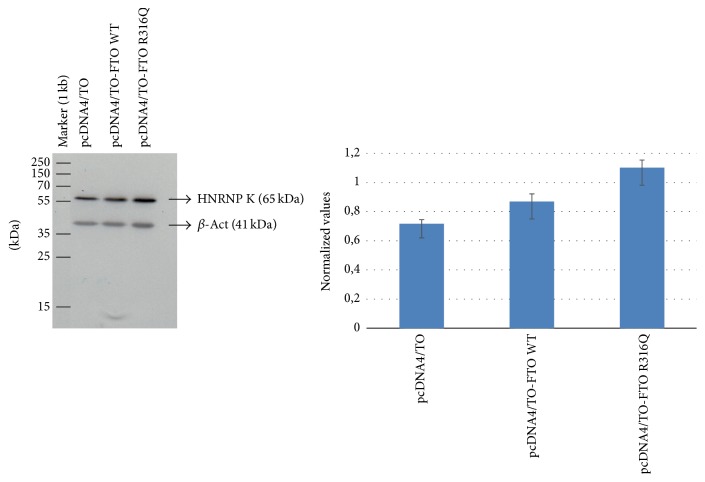
Western blot analysis of HNRNPK expression in pcDNA4/TO, the WT FTO, and the mutant (R316Q) FTO expressing cells. The bar graph was created using the intensity values of the bands obtained from ImageJ.

To monitor the changes observed at the proteome level well-resolved DIGE gels were generated from the soluble protein extracts prepared from FTO expressing and nonexpressing cells (Figure 4(a)). Analysis of DIGE images using spot detection tool revealed 350 (±20) spots per gel. Superimposition of the gel images over each other using PDQuest Advance revealed the presence of 11 differentially expressed proteins among the protein extracts. The regulated spots were excised from the preparative gels via ExQuest Spot cutter into a 96-well plate and successfully identified with MALDI-TOF/TOF analysis. Spots positions on the preparative gel are shown in Figure 4(b) with their corresponding SSP numbers. The identified proteins listed in Table 1 included Vimentin, Keratin Type II Cytoskeletal 1, Acetyl-CoA Acetyltransferase, Parathymosin, FTO, heterogeneous nuclear ribonucleoprotein K, Phosphoglycerate Kinase 1, and *α*-2-macroglobulin Receptor-associated Protein. To determine the level of regulation, we generated 3D images for each protein spot and the calculated spot intensities were used for comparisons (Figure 5). Statistical analysis with Student's* t*-test as implemented in PDQuest Advanced software yielded ratios for the identified proteins over the control (Table 2). Except two proteins, HNRNPK and Vimentin, the regulation ratios did not meet the 2-fold criteria. Therefore, to validate the differential expression of HNRNPK, western blot analysis was performed. The results indicated that the observed regulation represented the actual changes that occurred at the proteome level (Figure 6).

## 4. Discussion

In the present study, we investigated the possible effects of exogenously expressed FTO on the soluble proteome of 3T3-L1 cells. This cell line is one of the most robust cell culture models for adipogenesis and its relevance to FTO research has been confirmed [18]. Our purpose was to substantiate the existing theories on the FTO pathogenesis by way of expressing the WT and the mutant FTO proteins. We chose to study the R316Q mutant because it is the loss of function mutant of the FTO protein [12]. In FTO-deficient mice, this mutation causes severe malformations including postnatal growth retardation, significant reduction in adipose tissue, and lean body mass [19]. The purpose of expressing the mutant FTO protein was to repress the existing activity of endogenous FTO protein via increasing the quantity of the mutant FTO protein in the cells. In addition to the mutant protein, we expressed the WT FTO to enhance the activity of the existing endogenous FTO via increasing the quantity of the WT FTO proteins in the cell. The experimental approach (DIGE) allowed us to make comparisons among the WT FTO and the mutant FTO overexpressing cells over the control (the pcDNA4/TO transfected cells). Furthermore, we were able to mutually compare the protein profiles of the WT- and the mutant- overexpressing cells. Overall, there was a superior match among the protein profiles. When 2-fold regulation criteria were applied to the matching spots, three proteins came into view. One of those proteins was FTO itself indicated that any regulated protein spot could be caught by the experimental approach that was used in this study. This finding was also served as an internal control for the validity of the observed regulated protein spots. The other two proteins were Vimentin and HNRNPK. Petrak et al. (2008) have revealed in their meta-analysis that some proteins and protein families which are identified frequently as regulated in proteomic studies are regulated due to the stress caused by experimental conditions [20]. Interpretation of this suspicious regulation can cause misunderstanding of the results. Vimentin, which is listed as one of the frequently identified proteins, was, thus, disregarded in our further analysis. We focused our attention to HNRNPK which was upregulated by 3-fold over the control. What was interesting to see was that, in comparison to the control, HNRNPK was upregulated both in the WT and the mutant expressing cells. This has led us to propose that the regulation of HNRNPK was independent of the activity displayed or repressed by the FTO. In other words, the increase in HNRNPK level was irrespective of the activity enhanced by the WT FTO or repressed by the mutant FTO. The observed change in HNRNPK level may be reflected onto the FTO interactome. For example, if the increase in FTO level causes a saturation in FTO interaction partners such saturation may indirectly or directly cause an increase in the levels of proteins that are part of that interactome. We believe that HNRNPK is part of this interactome since its function is relevant to the physiological function of FTO in the cell. Indeed, both FTO and HNRNPK are part of the hnRNA metabolism. FTO oxidatively demethylates the adenine nucleotide on hnRNA while HNRNP binds this methylated adenine nucleotides [2, 21, 22].

**Figure 7:**
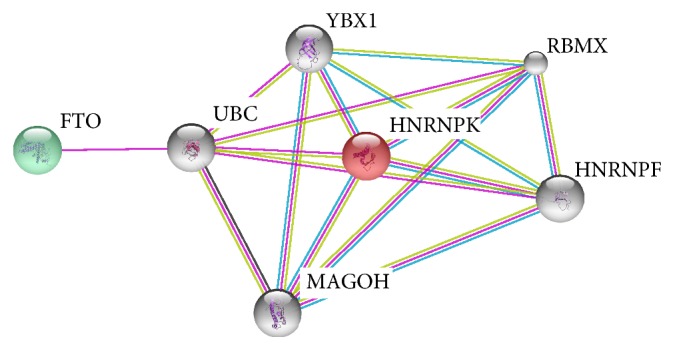
STRING analysis of HNRNPK and FTO interactions. The abbreviations stand for the following: FTO: fat mass and obesity-associated protein, HNRNPK: heterogeneous nuclear ribonucleoprotein K, UBC: ubiquitin C, YBX1: Y box binding protein 1, RBMX: RNA binding motif protein, X-linked, MAGOH: mago-nashi homolog, HNRNPF: heterogeneous nuclear ribonucleoprotein F.

HNRNPK is a subunit of heterogeneous ribonucleoprotein complex, which is a conserved RNA binding protein that is involved in multiple processes of gene expression, including processing of pre-mRNAs, mRNA export from the nucleus, RNA splicing, mRNA stability, and translation [23–25]. m6A is the most prevalent internal modification in hnRNAs in higher eukaryotes and widely conserved among eukaryotic species [26, 27]. This methylation of primer transcripts is catalyzed by METTL3-METTL14-WTAP heterotrimer structured methyltransferase complex [28]. Recent studies indicated that the methylation of hnRNA is a key code of hnRNA splicing and the mRNA translation. There are three different protein groups that control the translational regulation; writers, erasers, and readers. One of the erasers is FTO which mediates oxidative demethylation of m6A on RNA [28, 29]. Within this perspective, HNRNPK may be as the one of the translational regulator* (trans-factor)* and plays function as a reader on modified hnRNA. Like other readers, HNRNPK may be a part of the decision-making mechanism determining whether the transcript is going to be translated and at what level of quantity. Here, we suggested that there could be an indirect relationship between FTO and HNRNPK. To validate our prediction we performed STRING analysis using FTO and HNRNPK protein names. After retrieval of the experimental evidence from primary nonredundant interaction databases, STRING predicted that there is an indirect connection between FTO and HNRNPK proteins (Figure 7). This connection is achieved via ubiquitin C (UBC).

The increase of HNRNPK levels may seem very distant from the process typical of both FTO and UBC. However, the role of ubiquitin within the cell can be very complex and unique. Its functions extend from the well-known proteasome dependent proteolysis to cell signaling, transcription, apoptosis, and so on [30]. In addition, the effect of FTO expression on translation of certain proteins appears to be feasible. Zhou et al. demonstrated that a single m^6^A modification site in the 5′UTR enables translation initiation independent of the 5′-cap. The dynamic features of 5′UTR methylation expand the boundaries of physiological roles of FTO [31]. The details of such physiological roles are not understood to their full extend. Therefore, the parallel increase in expressions of FTO and HNRNPK may codictate the metabolic changes occurring at the protein level.

## Figures and Tables

**Table 1:** List of the identified regulated proteins and their respective MALDI-TOF/TOF data.

SSP numbers	Accession number	Best protein mass	Best protein score	Protein description	Expect	Observed	Mr (calc)	Ion score	Matched peptides	Sequence coverage (%)
1005	Q4FZU2	59213	56	Keratin, type II cytoskeletal 6A	0.059	1179,61	1178,593	50	K.YEELQITAGR.H	5

1602	Q4FZU2	668	88	Keratin, type II cytoskeletal 6A	4.2*e* − 005	1179,629	1178,593	80	K.YEELQITAGR.H	7

2502	P20152	53655	668	Vimentin	4,00*E* − 63	1296,635	1295,599	86	R.EEAESTLQSFR.Q	53
						1587,831	1586,79	85	R.TNEKVELQELNDR.F	
						1734,854	1733,808	76	R.LQDEIQNMKEEMAR.H	
						1254,588	1253,56	62	R.LGDLYEEEMR.E	
						1115,583	1114,562	53	K.VELQELNDR.F	
						2201,048	2199,974	47	R.EMEENFALEAANYQDTIGR.L	
						1093,54	1092,52	40	K.FADLSEAANR.N	
						1046,547	1045,523	28	K.LQEEMLQR.E	
	P48657	53424	192	Desmin	1.6*e* − 015	1587,831	1586,79	85	R.TNEKVELQELNDR.F	23
						1115,583	1114,562	53	K.VELQELNDR.F	

7203	Q8CAY6	41271	250	Acetyl-CoA acetyltransferase, cytosolic	2,50*E* − 21	1753,984	1752,952	69	K.AGHFDKEIVPVLVSSR.K	29
						2667,391	2666,312	50	K.VAPEEVSEVIFGHVLTAGCGQNPTR.Q	
						944,5446	943,5352	36	K.APHLTHLR.T	
						1935,019	1933,979	35	K.VNIDGGAIALGHPLGASGCR.I	
						1307,802	1306,797	23	R.ILVTLLHTLER.V	

7302	P55302	42189	230	Alpha-2-macroglobulin receptor-associated protein	2.5*e* − 019	1563,856	1562,83	69	K.IQEYNVLLDTLSR.A	36
						1823,859	1822,812	55	K.VSHQGYGSTTEFEEPR.V	
						1475,751	1474,716	26	K.HVESIGDPEHISR.N	
						1951,962	1950,907	16	R.KVSHQGYGSTTEFEEPR.V	

3602	P20152	53655	390	Vimentin	2.5*e* − 035	1587,822	1586,79	59	R.TNEKVELQELNDR.F	42
						1254,58	1253,56	59	R.LGDLYEEEMR.E	
						1296,628	1295,599	59	R.EEAESTLQSFR.Q	
						1444,728	1443,699	51	R.SLYSSSPGGAYVTR.S	
						1093,534	1092,52	47	K.FADLSEAANR.N	

1003	Q9D0J8	11423	69	Parathymosin	0.003	1389,627	1388,606	66	R.TAEEEDEADPKR.Q	11

1004	Q9C0B1	58245	427	Protein FTO	1,00*E* − 37	1914,94	1913,891	79	K.QGEEIHNEVEFEWLR.Q	45
						927,4879	926,4821	52	R.EGLPVEQR.N	
						1708,823	1707,779	50	K.MAVSWHHDENLVDR.S	
						1792,839	1791,792	44	K.ANEDAVPLCMSADFPR.V	
						1725,877	1724,84	36	R.VAECSTGTLDYILQR.C	
						1082,525	1081,509	28	R.QFWFQGNR.Y	

3603	P61979	50944	255	Heterogeneous nuclear ribonucleoprotein K	1.3*e* − 020	1194,702	1193,692	94	R.NLPLPPPPPPR.G	17
						1549,667	1548,645	63	K.LFQECCPHSTDR.V	
						1780,834	1779,791	55	R.TDYNASVSVPDSSGPER.I	
						1098,471	1097,445	23	K.GSDFDCELR.L	

4602	P61979	50944	316	Heterogeneous nuclear ribonucleoprotein K	6.4*e* − 028	1780,78	1779,791	82	R.TDYNASVSVPDSSGPER.I	25
						1194,677	1193,692	60	R.NLPLPPPPPPR.G	
						1098,432	1097,445	52	K.GSDFDCELR.L	
						1053,622	1052,634	45	R.VVLIGGKPDR.V	
						1549,63	1548,645	32	K.LFQECCPHSTDR.V	

7301	P09411	44522	277	Phosphoglycerate kinase 1	5,00*E* − 24	2782,383	2781,343	84	K.DCVGPEVENACANPAAGTVILLENLR.F	34
						1634,795	1633,785	66	K.LGDVYVNDAFGTAHR.A	
						1769,008	1767,988	39	K.ALESPERPFLAILGGAK.V	
						2023,034	2022,031	28	K.ITLPVDFVTADKFDENAK.T	

**Table 2:** Respective ratios for the regulated proteins over the control.

Protein name	R316Q/control	*p* values	WT/control	*p* values
Vimentin	5,75	0,0021	5,07	0,002
Acetyl-CoA acetyltransferase	0,865	0,03	0,675	0,025
Parathymosin	0,655	0,02	0,76	0,032
FTO	5,84	0,0019	5,73	0,002
Heterogeneous nuclear ribonucleoprotein factor K	3,775	0,002	2,6	0,003
Phosphoglycerate kinase 1	0,64	0,031	0,51	0,04
Α-2-macroglobulin receptor-associated protein	1,545	0,035	0,76	0,03
Keratin type II cytoskeletal 1	0,87	0,03	0,885	0,04
